# An Amphisbaenian Skull from the European Miocene and the Evolution of Mediterranean Worm Lizards

**DOI:** 10.1371/journal.pone.0098082

**Published:** 2014-06-04

**Authors:** Arnau Bolet, Massimo Delfino, Josep Fortuny, Sergio Almécija, Josep M. Robles, David M. Alba

**Affiliations:** 1 Institut Català de Paleontologia Miquel Crusafont, Universitat Autònoma de Barcelona, Cerdanyola del Vallès, Barcelona, Spain; 2 Dipartimento di Scienze della Terra, Università di Torino, Torino, Italy; 3 Department of Anatomical Sciences, Stony Brook University, Stony Brook, New York, United States of America; 4 FOSSILIA Serveis Paleontològics i Geològics, S.L., Sant Celoni, Barcelona, Spain; University of Florence, Italy

## Abstract

The evolution of blanid amphisbaenians (Mediterranean worm lizards) is mainly inferred based on molecular studies, despite their fossils are common in Cenozoic European localities. This is because the fossil record exclusively consists in isolated elements of limited taxonomic value. We describe the only known fossil amphisbaenian skull from Europe – attributed to *Blanus mendezi* sp. nov. (Amphisbaenia, Blanidae) – which represents the most informative fossil blanid material ever described. This specimen, from the Middle Miocene of Abocador de Can Mata (11.6 Ma, MN7+8) in the Vallès-Penedès Basin (Catalonia, NE Iberian Peninsula), unambiguously asserts the presence of *Blanus* in the Miocene of Europe. This reinforces the referral to this genus of the previously-known, much more incomplete and poorly-diagnostic material from other localities of the European Neogene. Our analysis – integrating the available molecular, paleontological and biogeographic data – suggests that the new species postdates the divergence between the two main (Eastern and Western Mediterranean) extant clades of blanids, and probably precedes the split between the Iberian and North-Western African subclades. This supports previous paleobiogeographic scenarios for blanid evolution and provides a significant minimum divergence time for calibrating molecular analyses of blanid phylogeny.

## Introduction

Amphisbaenians (worm lizards) constitute a poorly understood clade of burrowing and usually completely limbless squamates [1], [2]. Both molecular [3]–[8] and paleontological data currently indicate that amphisbaenians are the sister-taxon of lacertids, so that the former's limbless condition evolved independently from snakes. Amphisbaenians and lacertids probably diverged during the Late Cretaceous [9], although worm lizards are only undoubtedly recorded from the Paleogene onwards [10]. Among the 150–190 species of extant amphisbaenians [2], [11], most of them inhabit the southern continents (Afro-Arabia and South America), and only a few species are distributed in the Mediterranean region. Apart from *Trogonophis wiegmanni* (Trogonophidae), all extant Mediterranean amphisbaenians are included in the genus *Blanus* – previously allocated to the Amphisbaenidae, but currently included into a more basal family of their own, the Blanidae, both on the basis of molecular and morphologic evidence [2], [8], [12], [13].

The divergence of the various amphibaenian extant clades has been mainly related to vicariance events [5], [14]. Intercontinental oceanic dispersal events might have also occurred, as indicated by the purported sister-taxon relationship between the Mediterranean Blanidae and the Caribbean Cadeidae [13], although more recent results indicate that such relationship is uncertain [8]. With regard to Mediterranean worm lizards, molecular data consistently distinguish three extant clades of current disjunct distribution [15]: an Eastern Mediterranean clade (*Blanus strauchi*) [16]; an Iberian one (*Blanus cinereus*, and possibly the recently described cryptic species *Blanus mariae* – but see ref. [17]); and a North-Western African one (*Blanus mettetali* and *Blanus tingitanus*). Molecular evidence indicates that Iberian and North-Western African clades are more closely related to each other, with the Eastern Mediterranean clade having diverged first [12]. Molecular estimates of the divergence time among clades is mainly based on paleobiogeographic assumptions [12], due to the restricted information provided by the European fossil record of amphisbaenians, in spite of its relative abundance throughout the European Cenozoic [18].

The fossorial adaptations of amphisbaenians [2] are reflected in their cranial and postcranial osteology, thus facilitating their recognition in the fossil record, even if only disarticulated material is available. In Europe, the presence of a single family (Blanidae), at least regarding the Neogene, enables an easy identification at least at this level. Most findings consist in vertebrae or, more rarely, isolated tooth-bearing skull bones. The former, given the uniformity in postcranial anatomy of amphisbaenians [19], do not enable an attribution below the family level; the latter, in turn, display a rather uniform morphology from the Oligocene onwards (only members of Blanidae are represented) and provide restricted taxonomic information. Such a morphologic homogeneity, coupled with the high intraspecific variability inferred from some extant species, hinders the identification at the species level of most isolated fossil remains. The much more informative, but tiny and fragile, skulls of amphisbaenians are only rarely preserved. Thus, although some crania are known from the Cenozoic of North America [20]–[24] and Africa [25], in Europe a single cranial specimen from a putative stem amphisbaenian is known from the Eocene [9]. This preservational bias explains why, for extinct blanids, only three species of two different genera are currently recognized (on the basis of lower jaws): *Palaeoblanus tobieni*, from MP27-MN13 of France, Germany, Italy and Spain [26]–[28]; *Blanus antiquus*, from the MN3–MN6 of Austria and Germany [29]; and *Blanus gracilis*, from the MN2–MN4 of the Czech Republic and Italy (and, with doubts, from the MN7+8 of Romania) [30]–[32].

Here we describe a new species of *Blanus*, based on an exceptionally preserved, complete skull and numerous vertebrae from a single Middle Miocene locality of the Vallès-Penedès Basin (NE Iberian Peninsula). The described cranial specimen, which represents the first fossil blanid skull thus far described, sheds new light on the evolution of Mediterranean worm lizards.

### Age and geological background of the type locality of *Blanus mendezi* sp. nov

The fossil remains described in this paper come from Abocador de Can Mata (ACM) [33]–[35]. This stratigraphic series is situated in the Vallès-Penedès Basin (NE Iberian Peninsula) – a NNE-SSW-oriented half-graben limited by the Littoral and Pre-littoral Catalan Coastal Ranges, which was generated by the rifting of the NW Mediterranean region during the Neogene [36]–[39]. Except for some Early and Middle Miocene shallow marine and transitional sequences, most of the basin infill consists of marginal alluvial fan sediments with a rich fossil record of Early, late Middle and Late Miocene terrestrial vertebrates [40], [41].

ACM localities are situated in the area of els Hostalets de Pierola, which displays thick Middle to Late Miocene alluvial sequences. They were deposited in distal-to-marginal, inter-fan zones of the coalescing alluvial fan systems of els Hostalets de Pierola and Olesa [42]. More than 250 localities have been defined along the ACM composite series (ca. 250 m in thickness), which can be accurately dated based on lithostratigraphic, magnetostratigraphic and biostratigraphic correlation [34], [35], [41], [43]. The whole series spans from ca. 12.5 to 11.4 Ma [43], whereas locality ACM/C8-A4 (from which all the remains reported in this paper come from) is correlated to subchron C5r.2 n, with an interpolated age of 11.6 Ma (late Aragonian, close to the Middle to Late Miocene boundary).

## Materials and Methods

### Permits

No permits were required to carry out this study, since the described fossil specimens (see catalog numbers below) are adequately curated at Institut Català de Paleontologia Miquel Crusafont. The fossils were recovered by Josep M. Méndez, a technician of this institution, by screen-washing sediments previously excavated in 2011, in the course of a paleontological excavation directed by one of the authors (Josep M. Robles), under a permit (437 K121 N352 2011-1/6509) issued by the Servei d'Arqueologia i Paleontologia of the Generalitat de Catalunya (Catalan local government).

### Locality and institutional abbreviations

ACM, local stratigraphic series of Abocador de Can Mata; C8, Cell 4 of ACM; AMNH, American Museum of Natural History (New York, USA); DP FNSP, Department of Palaeontology, Charles University, Prague (Czech Republic); ICP, Institut Català de Paleontologia Miquel Crusafont, Universitat Autònoma de Barcelona (Spain); IPS, collections from the ICP; MDHC, Massimo Delfino's Herpetological Collection, housed at the Dipartimento di Scienze della Terra, Università di Torino (Italy).

Note that terminology is based on [44] and abbreviations used in figures can be found in corresponding figure captions, but an alternative terminology exists [45], and has been used where indicated.

### Computed tomography

IPS60464 was scanned on a GE phoenix v|tome|x s180 (GE Measurement & Control Solutions, Hanover, Germany) at the American Museum of Natural History (AMNH) using a nanofocus X-ray tube with the following parameters: voltage 105 kV and current 70 mA and a magnification of 15.86723491. We obtained 1100 slices with slice thickness of 0.2 mm and a pixel size of 0.01260459 mm. The raw data were imported to VG Studio Max 2.1 and exported to Avizo 7.0 for analysis, segmentation, and visualization. We segmented each bone slide by slide and deleted the covering crust and the infilling matrix present in the original fossil by considering the different densities of bone, crust and sediment in Avizo 7.0.

### Material

The accessed recent specimens include mostly disarticulated skulls and vertebrae of one *Blanus cinereus* (MDHC 156) and three *B. strauchi* (MDHC 286-8), as well as articulated and disarticulated material of both species from the personal collection of S. Bailon (MNHN, Paris). Fossil amphisbaenians have been accessed at the MNHN and the ICP.

### Nomenclatural Acts

The electronic edition of this article conforms to the requirements of the amended International Code of Zoological Nomenclature, and hence the new names contained herein are available under that Code from the electronic edition of this article. This published work and the nomenclatural acts it contains have been registered in ZooBank, the online registration system for the ICZN. The ZooBank LSIDs (Life Science Identifiers) can be resolved and the associated information viewed through any standard web browser by appending the LSID to the prefix “http://zoobank.org/”. The LSID for this publication is: urn:lsid:zoobank.org:pub: 062AC1C9-86C7-4271-B7A4-056F1DBA52A4. The electronic edition of this work was published in a journal with an ISSN, and has been archived and is available from the following digital repositories: PubMed Central, LOCKSS.

## Systematic Paleontology

Order Squamata Oppel, 1811.

Suborder Amphisbaenia Gray, 1844.

Family Blanidae Kearney, 2003.

Genus *Blanus* Wagler, 1830.

### *Blanus mendezi* sp. nov

Nomenclatural statement: An LSID number was obtained for the new taxon (*Blanus mendezi* Bolet et al.): urn:lsid:zoobank.org:act: 8433DC0F-A209-4F5F-8F8A-B97C48BF93EB.

**Figure 1:**
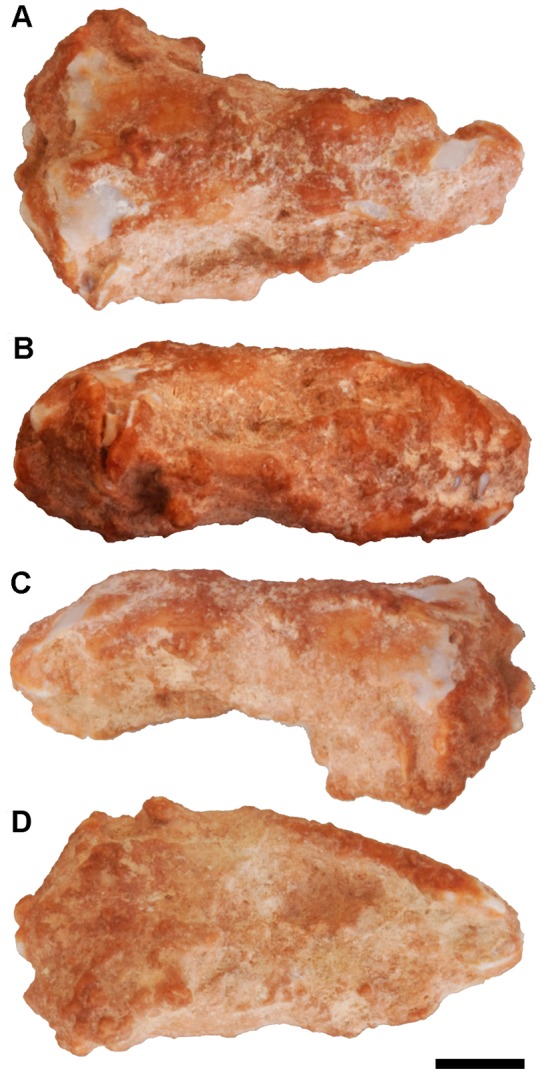
*Blanus mendezi* sp. nov. IPS60464 as preserved. Holotype in (A) dorsal, (B) right lateral, (C) left lateral and (D) ventral views. Scale bar equals 2 mm.

**Figure 2:**
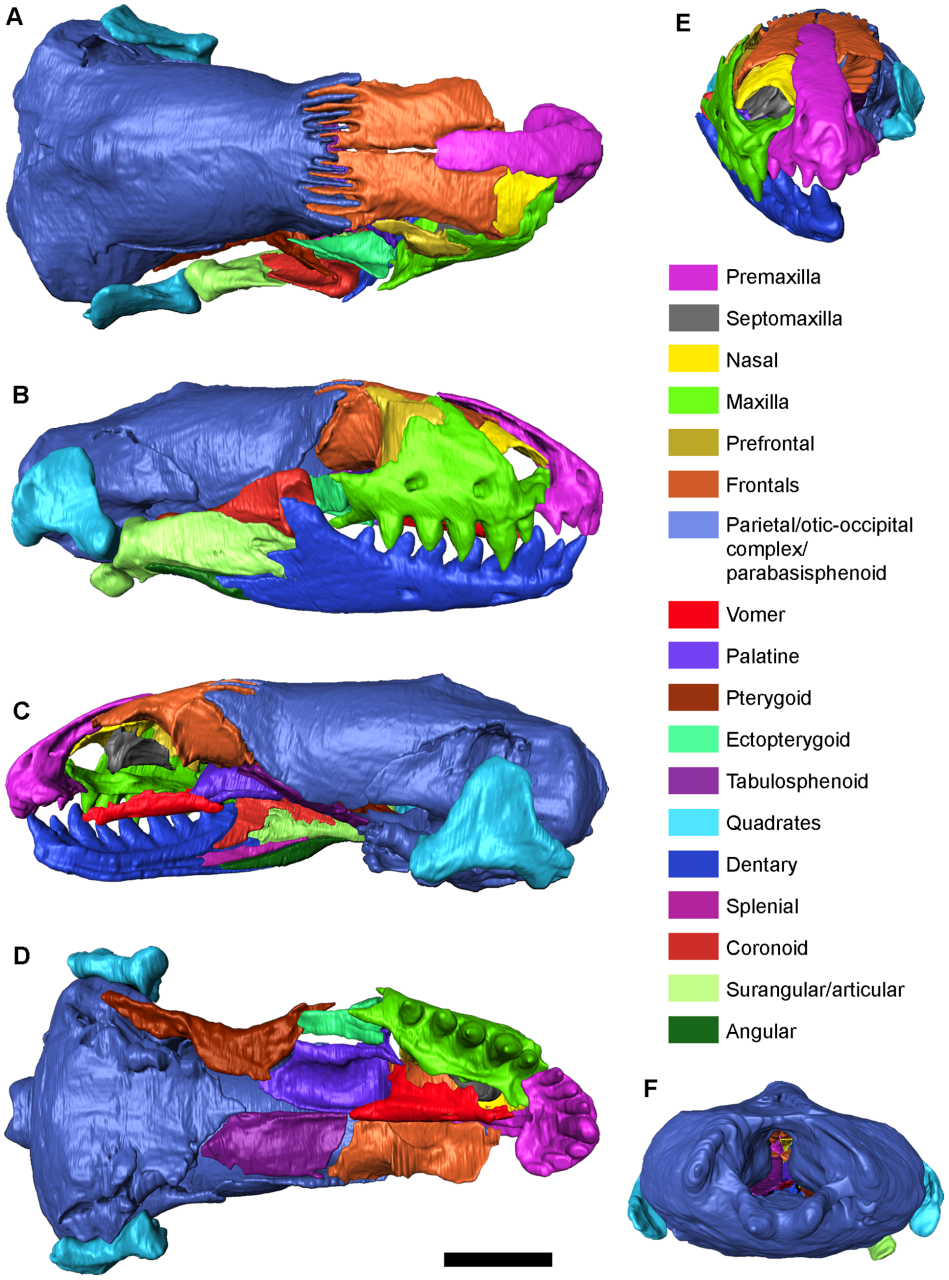
*Blanus mendezi* sp. nov., virtual model of the holotype (IPS60464) after removing the covering crust and the infilling matrix. Model in (A) dorsal, (B) right lateral, (C) left lateral, (D) ventral and (E) anterior and (F) posterior views. Scale bar equals 2 mm.

**Figure 3:**
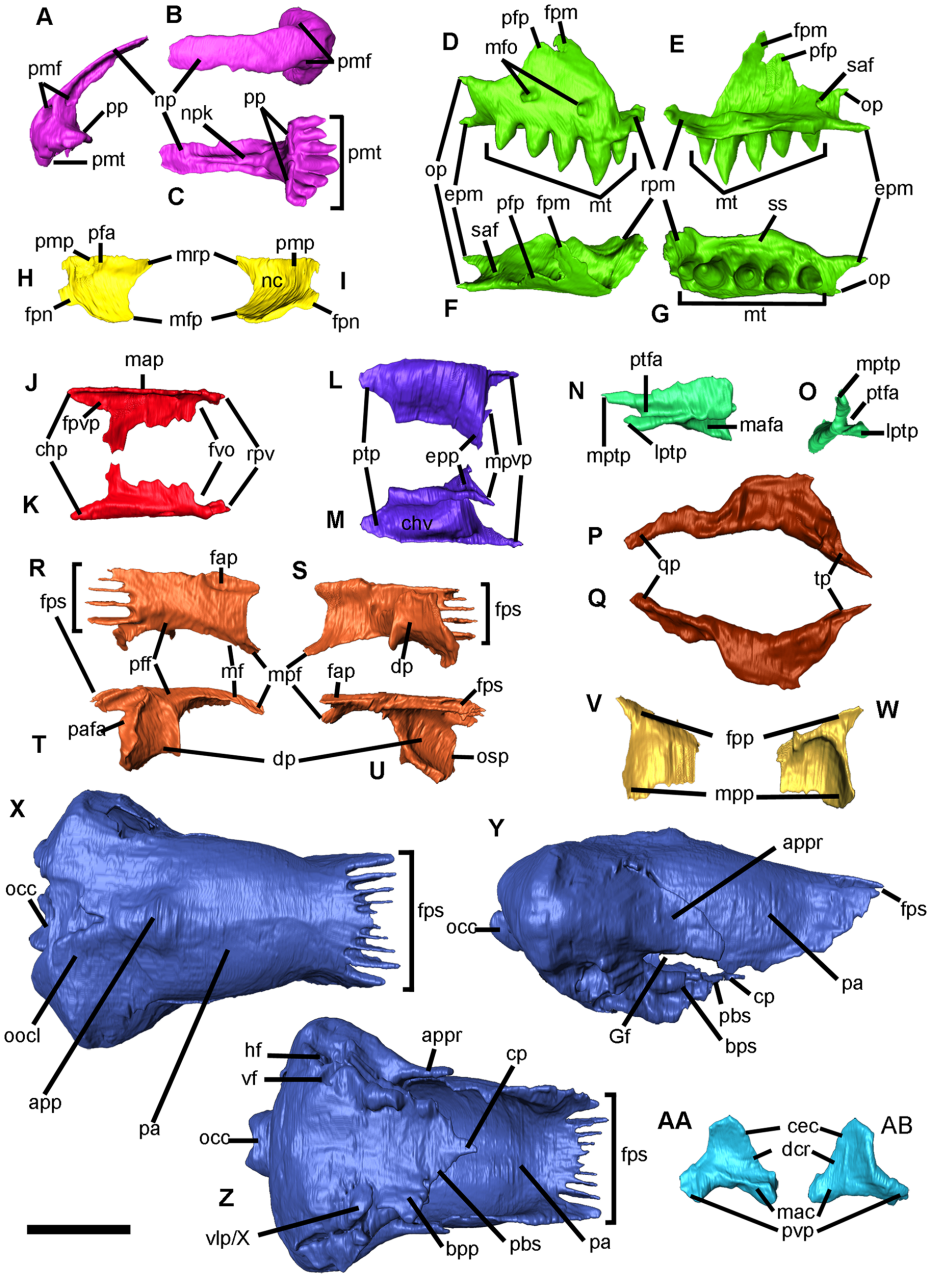
*Blanus mendezi* sp. nov., virtual model of selected skull bones of the holotype (IPS60464). (A–C) Premaxilla in left lateral (A), dorsal (B), and ventral (C) views. (D–G) Right maxilla in labial (D), lingual (E), dorsal (F) and ventral (g) views. (H, I) Right nasal in dorsal (H) and ventral (I) views. (J, K) Right vomer in dorsal (J) and ventral (K) views. (L, M) Right palatine in dorsal (L) and ventral (M) views. (N, O) Right ectopterygoid in labial (N) and anterior (O) views. (P, Q) Right pterygoid in dorsal (P) and ventral (Q) views. (R–U) Right frontal in dorsal (R), ventral (S), lateral (T) and medial (U) views. (V, W) Right prefrontal in lateral (V) and medial (W) views. (X–Z) Parietal/otic-occipital complex/parabasisphenoid in dorsal (X), right lateral (Y) and ventral (Z) views. (AA, AB) Left quadrate in lateral (AA) and medial (AB) views. Colors correspond to those in figure 1. Abbreviations: app, apical process of parietal; appr, alar process of prootic; bps, basipterygoid process; cec, cephalic condyle of quadrate; chp, choanal process of vomer; chv, choanal vault; cp, cultriform process of parabasisphenoid; dcr, dorsal crest of quadrate; dp, descending process of frontal; epm, ectopterygoid process of maxilla; epp, ectopterygoid process of palatine; fnpp, frontal facet for the nasal process of premaxilla; fpm, frontal process of maxilla; fpn, frontal process of nasal; fpp, frontal process of prefrontal; fps, frontoparietal suture; fvo, fenestra vomeronasalis; fvp, facet for palatine vomerine process; Gf, Gasserian foramen; hf, hypoglossal foramen; lptp, lateral pterygoid process of ectopterygoid; mac, mandibular condyle of quadrate; mafa, ectopterygoid facet for the articulation of the ectopterygoid process of maxilla; map, median articular plane; mfo, maxilla labial foramina; mfp, maxillary facial process of nasal; mp, maxillary process of palatine; mpf, maxillary process of frontal; mpp, maxillary process of prefrontal; mrp, maxillary rostral process of nasal; mt, maxillary teeth; mptp, medial pterygoid process of ectopterygoid; mf, frontal facet for maxilla and prefrontal; nc, nasal chamber; np, nassal process of premaxilla; npk, nasal process of premaxilla keel; occ, occipital condyle; oocl, otic-occipital lapet; op, orbital process of maxilla; osp, ventral process of frontal; pa, parietal; paf, frontal facet for parietal; pfa, facet of frontal for the nasal process of premaxilla; pbs, parabasisphenoid; pff, frontal facet for prefrontal; pfp, prefrontal process of maxilla; pmf, premaxilla foramina; pmp, premaxillary process of nasal; pmt, premaxillary teeth; pp, palatal process of premaxilla; ptfa, ectopterygoid facet for pterygoid; ptp, pterygoid process of palatine; pvp, posteroventral process of quadrate; qp, quadrate process of pterygoid; rpm, rostral process of maxilla; rpv, rostral process of vomer; saf, superior alveolar foramen; ss, supradental shelf of maxilla; tp, transverse process of pterygoid; vf, vagus foramen; vlp/X, ventrolateral process/“element X”; vp, vomerine process of palatine. Scale bar equals 2 mm.

**Figure 4:**
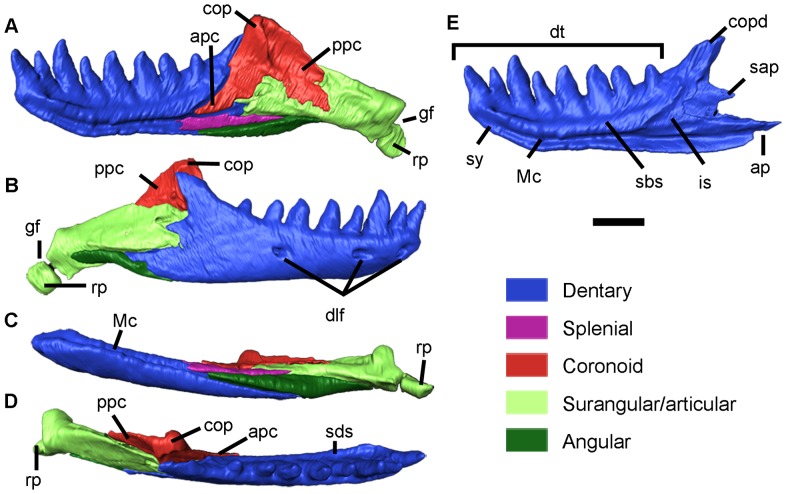
Virtual model of the lower jaw of *Blanus mendezi* sp. nov., based on the holotype (IPS60464). (A–D) Lower jaw, in lingual (A), labial (B), ventral (C) and dorsal (D) views. (E) Dentary in lingual view. Abbreviations: ap, articular process of dentary; apc, anterior process of coronoid; cop, coronoid process of coronoid; copd, coronoid process of dentary; dlf, dentary labial foramina; dt, dentary teeth; gf, glenoid fossa; is, intramandibular septum; Mc, Meckelian canal; ppc, posterior process of coronoid; rp, retroarticular process; sap, surangular process; sbs, subdental shelf of dentary; sy, symphysis. Scale bar equals 1 mm.

Holotype: IPS60464, complete skull (cranium with articulated lower jaw; Figs. 1–4, and Video S1), housed at the ICP.

Paratypes: IPS63989, four cervical vertebrae in anatomical connection (Fig. S1A–D); IPS63990, trunk vertebra (Fig. S1E–I); IPS63991, trunk vertebra (Fig. S1J–N); IPS63992, trunk vertebra (Fig. S1O–S); IPS63993, trunk vertebra (Fig. S1T–X); IPS63995, 40 trunk vertebrae.

Type locality: ACM/C8-A4, 11.6 Ma (Middle Miocene), Catalonia, Spain.

Etymology: dedicated to ICP technician Josep M. Méndez, who found the holotype while carefully picking up microvertebrate remains.

### Diagnosis

Large-sized species of *Blanus* with a slightly protruding snout. Dentition heterodont, with robust pleurodont teeth (seven premaxillary, five maxillary, eight dentary), the first dentary tooth being smaller than the third one. Tooth-bearing bones robust. Nasal process of the premaxilla long. Frontals long relative to the skull, with an almost straight suture between them, and a well-developed facet for articulation with the maxilla and the prefrontal; frontoparietal suture strongly interdigitated. Long, acuminated and medially-directed orbital process present in the maxilla. Premaxilla anteriorly (not ventrally) projected. Cervical and anterior trunk vertebrae with paracotylar tubercles.

### Differential diagnosis

Regarding extinct taxa, the new species differs from *B. antiquus* in the larger size and more heterodont dentition (greater variability in the height and robustness of the teeth); and from *B. gracilis*, in the much larger size, the more robust tooth-bearing bones and teeth, and the more closely packed teeth. The new species also differs from all extant *Blanus* spp. in the larger size and – as far as it can be ascertained for those species for which cranial osteology is known (*B. cinereus* and *B. strauchi*) – in the longer nasal process of the premaxilla, the relatively longer frontals compared to the rest of the skull, the more straighter suture between the frontals, the more developed frontal articular facet for the maxilla and prefrontal, and the presence of a longer and posterodorsally directed maxillary orbital process. Additionally, the new species further differs from *B. strauchi* in the less protruding snout lacking a ventrally-projected proximal tip of the premaxilla, as well as in the stouter teeth; and from both *B. cinereus* and *B. strauchi*, in the stronger interdigitation of the frontoparietal suture. The paracotylar tubercles of the cervical and trunk vertebrae are unknown in the rest of *Blanus* spp., but a similar structure might be present in *B*. *gracilis*.

## Description

IPS60464 is an almost complete skull (11.3 mm in length) that includes the right lower jaw in articulation (Figs. 1 and 2). The specimen is exceptionally well preserved, including all unpaired elements, whereas all paired bones are represented at least in one side (Figs. 2 and 3). The skull is however covered by a carbonate concretion that obscures most of its external morphology. It also displays a matrix infilling that precludes the observation of the palate, the inner surfaces of the skull roof and the lingual surfaces of the lower jaw. The small size and fragility of the specimen precluded mechanical preparation, so its description is based on computed tomography (CT) scans. The latter not only revealed the external morphology, but further granted access to the internal cranial morphology (otherwise unobservable), thereby enabling the description of isolated bones and their joint surfaces. A description of the skull and vertebrae (Fig. 2 and Fig. S1) is provided below, followed by comparisons with fossil and extinct blanids.

**Figure 5:**
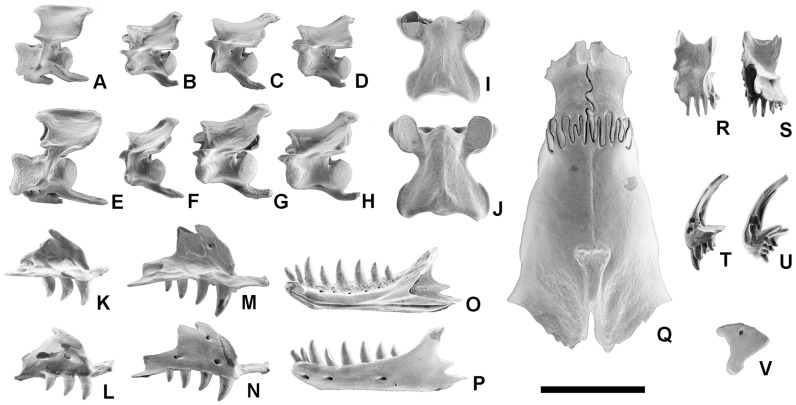
Selected material of extant *Blanus cinereus* (MDHC 156) and *Blanus strauchi* (MDHC 286) for comparison purposes. (A–H) Cervical vertebrae of *B*. *cinereus* (A–D) and *B*. *strauchi* (E–H), in left lateral view. (I–J) Dorsal vertebrae of *B*. *cinereus* (I) and *B*. *strauchi* (J), in dorsal view. (K–N) Left maxillae of *B*. *cinereus* (K) and *B*. *strauchi* (M), in lingual view; right maxillae of *B*. *cinereus* (L) and *B*. *strauchi* (N), in labial view. (O–P) Left dentary of *B*. *strauchi*, in lingual (O) and labial (P) views. (Q), Articulated parietal and frontals of *B*. *strauchi*, in dorsal view. (R–S) Frontals of *B*. *strauchi*; right frontal in dorsal view (R), and left frontal in ventral view (S). (T–U) Premaxillae of *B*. *cinereus* (T) and *B*. *strauchi* (U), in left lateral view. (V) Left quadrate, in lateral view. Scale bar equals 2 mm.

**Figure 6:**
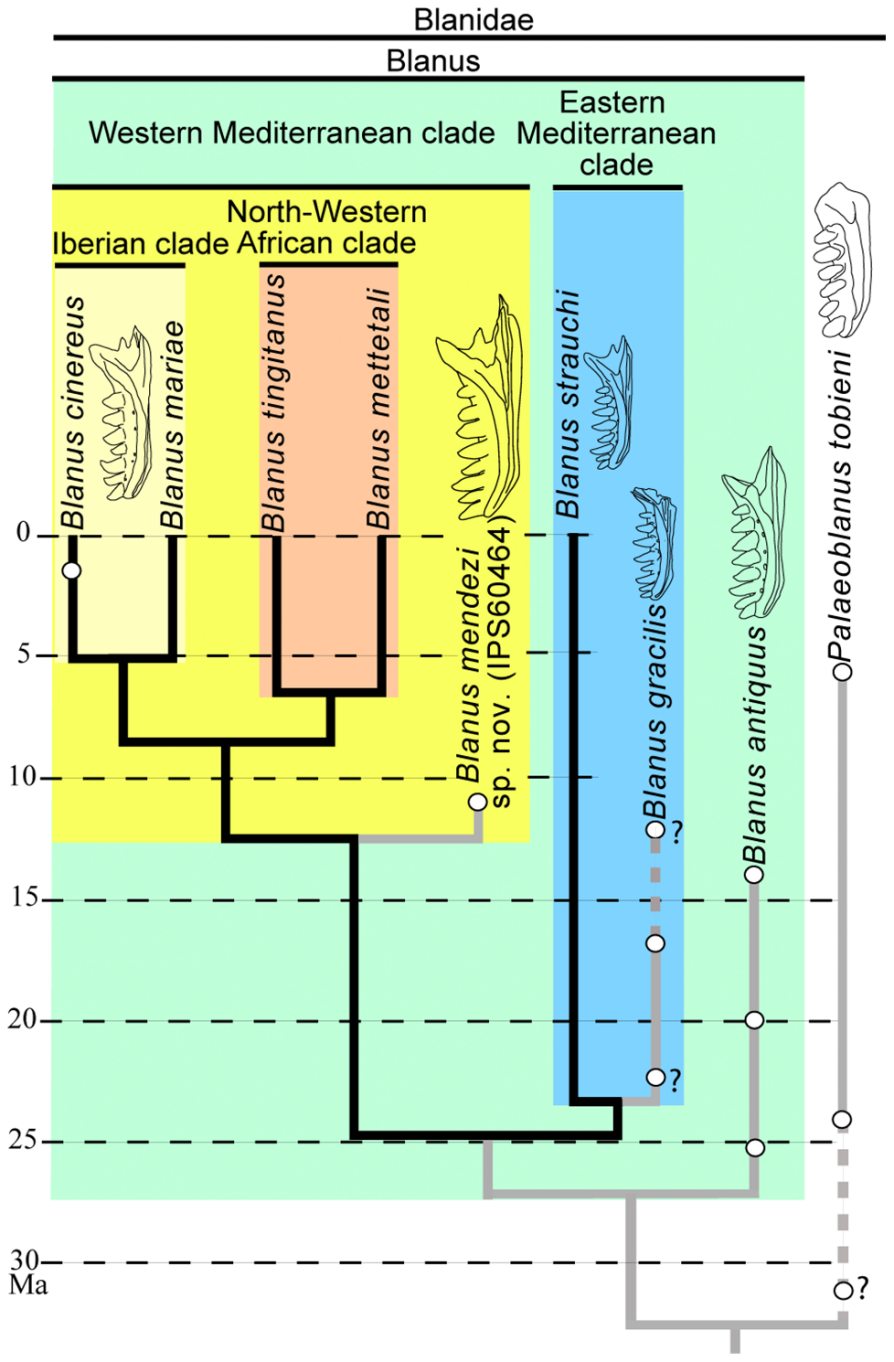
Evolutionary tree of the Blanidae based on molecular phylogeny, paleobiogeographic evidence and the paleontological data discussed in this paper. Black branches depict the phylogeny and estimated divergence times for extant taxa based on molecular data [12], [49]; grey branches, in turn, depict inferred stratigraphic ranges (dashed when uncertain) based on fossil finds and divergence times, as well as the hypothesized branching order for extinct species, based on morphology and biogeography e.g. [16], [52], [87]. *Blanus cinereus* and *B*. *antiquus* redrawn from ref. [29]; *B*. *gracilis* redrawn from ref. [30]; *B*. *strauchi* redrawn from ref. [88]; *Palaeoblanus tobieni* redrawn from ref. [50].

IPS60464 bears seven premaxillary (Fig. 3C), five maxillary (Fig. 3D) and eight dentary (Fig. 2 and 4A) pleurodont teeth; the first dentary tooth is smaller than the third one, as in other species of *Blanus*. The teeth are robust (comparable to *B. cinereus*, *B. antiquus* and *P. tobieni*), contrasting with the much more slender dentition of *B*. *gracilis* and *B*. *strauchi* (Figs. 5 and 6). The premaxilla bears a very long, apically truncated nasal process (Fig. 3A–C), and the snout is only weakly protruding – similar to that of *B*. *cinereus* (Fig. 5T) and fossil forms, but contrasting with the more clearly protruding snout with a ventrally-directed premaxilla of *B*. *strauchi* (Fig. 5U). The frontals are relatively long (3.1 mm) and roughly rectangular (Fig. 3R–U), with an almost straight suture between them and a strong interdigitation with the parietal (Fig. 2A). The nasals are short relative to the frontals (Fig. 2A). The maxilla has a medially directed rostral process (Figs. 2B and 3F) and an unusually long and pointed orbital process (figure 3D–G). The prefrontal is present and well developed (Figs. 2A and B, and 3V and W), precluding the contact between the maxilla and frontal except in the anterior lateral margin of the latter (Fig. 2A). Elements of the palate (Fig. 3J–Q) are observable, but at present provide little taxonomic information because their morphology in other taxa is barely known. The parietal (Figs. 2A–C and 3X–Z) is by far the largest bone of the skull; although its limits with the otic-occipital complex (Figs. 2B and 3Y and Z) are clear in some regions, we were unable to completely separate them due to partial fusion or, more likely, limitations in the resolution of the CT-scan. The cranial proportions of IPS60464 roughly fit those reported for extant species – only described for *B*. *cinereus* and *B*. *strauchi* [46], [47], [48], [49] – except for the relatively shorter preorbital region displayed by the fossil specimen (ca. 25%, in front of 30% in the two morphotypes of *B*. *cinereus*, see Fig. S2). The quadrate (Figs. 2, and 3AA and AB) is rather robust. The cranium of the new species (length, 11.3 mm; width, 5.8 mm) is larger (ca. 25% longer) than that of all extant blanids [48]. The comparison of the dimensions of isolated tooth-bearing bones also indicate for *B. mendezi* a slightly larger size than for *B*. *antiquus* and *P*. *tobieni*, and a much larger size than for *B*. *gracilis* (Fig. 6).

The lower jaw (Figs. 1B and 3A–D) displays the typical blanid configuration [2]; the dentary (Fig. 4E), due to its heterodonty, is clearly distinct from those of both *Palaeoblanus tobieni* and *Blanus antiquus*, which display a homodont dentition (see figures in refs. [29], [50]) mainly regarding tooth height and robustness. The dentary of IPS60464 is much larger than those of *B*. *gracilis*, *B*. *strauchi* and *B*. *cinereus*, but only slightly larger than those of *B*. *antiquus* and *P*. *tobieni* (Fig. 6).

Both the neck and anterior trunk vertebrae (Fig. S1) show the typical amphisbaenian morphology (i.e., dorsoventrally flattened and without neural spine) (e.g. [51]). They are however further characterized by the presence of paracotylar turbercles, which are unknown from other *Blanus* spp. The largest vertebrae of the new species, in agreement with skull size, are slightly larger than the largest Neogene *Blanus* vertebrae reported so far [50], [51], also much larger than those of extant species – at least regarding *B*. *cinereus* and *B*. *strauchi* (AB pers. obs.), since these are unknown for *B*. *mettetali* or *B*. *tingitanus*, although these two species are reported to be smaller than *B*. *cinereus* [52].

### Extended description of the skull

#### Cranium

The cranium measures 11.3 mm from the tip of the snout to its most posterior projection (Figs. 1, 2), 5.8 mm of maximum width at the posterior region of the parietal, and 3.9 mm of maximum height. The preorbital region measures 3 mm, representing 26% of the total length.

The azygous premaxilla (Fig. 3A–C) bears seven tooth positions; the central one is greatly enlarged, and all of them are robust and cylindrical. This is evident even considering the poor preservation of the central tooth and the right lateral teeth being broken at different levels. It is not possible to discern whether the lateral teeth were much shorter than the others, but a moderate decrease in size is suggested by the CT sections. The nasal process of the premaxilla is broad and very long, slightly tapering dorsally and with a subtle waisting at its base. The inner surface of the nasal process is provided with a prominent and long medial keel (Fig. 3C). The anterior external surface is pierced by two large foramina having their exit on the inner side (longitudinal canal of [45]). The nasal process of the premaxilla precludes the dorsal contact between the nasals and that of the frontals in their anterior third (Fig. 2A). The poorly developed palatal process laterally contacts the rostral process of the maxilla. The palatal process probably contacted the vomer in its original position, but displacement or incomplete preservation of the latter results in the lack of contact in the fossil (Fig. 2D). The supradental platform is horizontal and thin, and displays a central notch.

The right septomaxilla (Fig. 2C) is present and appears rather simple in structure, although it should be taken into account that some processes formed by thin bone may have been either not preserved or artificially deleted during the CT-scan processing. This is supported by the fact that the septomaxilla does not contact the surrounding bones, whereas it should be in contact with the premaxilla, maxilla and/or nasal. The general ventrally convex shape of the septomaxilla, however, agrees with that of *Blanus cinereus* according to the material figured in the literature [47] and examined in the comparative sample.

The maxilla (Figs. 2, 3D–G), only preserved on the right side, bears five robust and only weakly curved teeth, the second one being the largest, and the first one the smallest. The reduction of the first maxillary and the most lateral premaxillary teeth allows for the necessary space to accommodate the enlarged third dentary tooth when the mouth is closed (Fig. 2B). Distalwards from the second tooth, there is a reduction in maxillary tooth height. The supradental shelf is wide, and the sulcus dentalis is apparently lacking or only slightly developed (Fig. 3G). The superior alveolar foramen is situated at the level of the distal margin of the last tooth (Fig. 3E). The maxilla contacts the premaxilla and the maxillary rostral process of the nasal through a rather wide and medially directed rostral process (premaxillary process of the maxilla in [45]) (Fig. 3D–G), as well as the frontal and prefrontal bones in its dorsal and posterior margins, respectively (Fig. 2A, B). The orbital process, situated dorsolabially, is relatively long (Fig. 3D–G) compared to other *Blanus* species. The dorsal process (frontal process of [45]) approaches the bifurcated condition seen in trogonophids [53] as well as *B. cinereus* and *B*. *strauchi* (Fig. 5K–N), although in the former the prefrontal is absent [44]. The maxilla has a long posteroventrally positioned process (ectopterygoid process; Fig. 3D–G), which lies ventrolaterally to the anterior extension of the ectopterygoid. Two large foramina pierce the maxilla at the level of the posterior edge of the second and fourth tooth (Fig. 3D).

The nasal (Figs. 2A, B and 3H, I), only preserved on the right side, is large and trough-shaped. A medial deep facet receives the nasal process of the premaxilla (Fig. 3H, I). Anteriorly, a ventrally-directed process approaches the maxillary rostral process, whereas a less developed and laterally-positioned process contacts the dorsal margin of the maxilla (Fig. 3H, I). Its anterior margin is truncated, sensu ref. [2]. Posteriorly, the frontal process lies ventrally to the frontal (Fig. 3H, I). The snout is rounded, and the naris (formed by the premaxilla, the nasal and the maxilla) opens anterodorsally (Fig. 2A, B, E). The protrusion of the snout is only weakly developed.

The palate is preserved on the right side, formed by the palatal processes of the premaxilla, the supradental shelf of the maxilla, the vomer, the palatine, the ectopterygoid and the pterygoid (Fig. 2D). Teeth are absent from the palate (pterygoid, vomer and palatine are edentulous; Fig. 2D). The paired vomer is elongate, with a straight median articular plane and a poorly developed rostral process (note that this could be a preservational or CT-scan artifact) that surrounds the anterior margin of a small fenestra vomeronasalis (Figs. 2D and 3J, K). Posteriorly, this bone displays a facet for the articulation of the vomerine process of the palatine and the posteriorly directed choanal process (Fig. 3J, K). The paired palatine (Figs. 2D and 3L, M) is wide and its inferior surface is highly arched, forming the roof of the choanal vault. It bears a vomerine process anteromedially, a maxillary process anterolaterally, and a pterygoid process laterally; its lateral margin runs parallel to the ectopterygoid. Posteriorly, it displays a roughly triangular pterygoid process (Fig. 3L, M), which contacts the transverse process of the pterygoid (Fig. 2D). The paired ectopterygoid (Fig. 3N, O) bears a forked anterior maxillary process; laterally, the latter process bears a facet for the maxillary facial ectopterygoid process (Fig. 3O), as well as a posterolaterally-situated, deep and narrow facial facet for the articulation with the transverse process of the pterygoid, which is clasped by two (dorsomedial and ventrolateral) processes (Fig. 3N). The paired pterygoid (Figs. 2D and 3P, Q) articulates with the ectopterygoid and the palatine anteriorly, with the parabasisphenoid medially, and with the quadrate posteriorly (Fig. 2D). The pterygoid has a very long and slender posterior region, with a poorly defined quadrate process, whereas it suddenly widens anteriorly, where it forms a platform that contributes to the palate (Figs. 2D and 3P, Q). The transverse process is laterally directed and receives the pterygoid process of the ectopterygoid (Figs. 2D and 3P, Q).

The frontals are paired, with a rather straight suture between them, and a strongly interdigitated suture with the parietal (Figs. 2A and 3R–U). These bones are almost three times longer than wide, and the long nasal process of the premaxilla precludes the dorsal contact between the two frontals for at least one third of their length (Figs. 2A and 3R, U). However, the frontals are in contact below the nasal process of the premaxilla, and have a well-marked facet to receive it (Fig. 3R, U). Posteroventrally, they show strong and ventrally-directed (descending) processes (Fig. 3S–U), which meet each other in the midline and contact the tabulosphenoid posteriorly (Fig. 2D). The suture between the frontal and the nasal, which has been slightly displaced below the frontal, is arched (Fig. 2A). The frontal contacts the maxilla, separating the large prefrontal from the nasals (Fig. 2A, B). The dorsolateral surface of the frontals bears a marked facet for articulation with the maxilla and prefrontal (Figs. 2A and 3R).

The paired prefrontal (Figs. 2A, Band 3V, W), only preserved on the right side, precludes the contact between the maxilla and the frontals only in the posterior-most portion of the former. The prefrontal has a posterodorsally-directed, pointed frontal process as well as a wider, ventrally-directed maxillary process (Fig. 3V, W).

The unpaired parietal (Figs. 2A–C and 3X–Z) is long, more than twice the length of the frontals. It displays a dorsal protuberance (Figs. 3X) that marks the beginning of what might represent an incipient sagittal crest – in fact, the latter is observable in the CT sections, in spite of not being clearly expressed on the surface. The lateral walls of the parietal are vertically developed, being closed by the frontals anteriorly, the tabulosphenoid (sensu [54], orbitosphenoid of [44]) anteroventrally, the parabasisphenoid ventrally, and the otic-occipital complex posteroventrally (Figs. 2C, D and 3Y).

The occipital condyle is bicipital, and connects to the basioccipital plate through a rather wide neck. The foramen magnum is bordered by the exoccipitals and supraoccipital, the latter presenting a wide dorsoposteriorly positioned notch almost reached by the posterior margin of the parietal. The alar process of the prootic is rather long, and the paroccipital processes are laterally oriented. Although some additional elements have been identified (e.g. vagus foramen, ventrolateral process/“element X”, hypoglossal foramen), the description of their morphology is precluded by the poor preservation of the region and/or a lack of resolution of the CT-Scan.

The orbit is formed by a small anterior portion of the parietal and the tabulosphenoid, the lateral margin of the frontal, the prefrontal, a small posterior portion of the maxilla and the dorsal margin of the ectopterygoid (Fig. 2A, B). The tabulosphenoid only preserved on the left side, is a paired (or unpaired but broken in its midline) element situated dorsally from both the palatine and pterygoid; it contacts anteriorly with the posteroventral margin of the descending process of the frontal (Fig. 2C, D). It is possible that the parabasisphenoid is co-ossified (Figs. 2D and 3Z), although this bone sometimes appears disarticulated in *Blanus* specimens (this could also be related to a younger ontogenetic age of the accessed specimens). The orbital rim is incomplete posteriorly, due to the lack of a jugal (Fig. 2B).

Both quadrates are preserved (Figs. 2A–F and 3AA, AB), the right one in articulation with the lower jaw (Fig. 2A, B). They are robust, and their dorsal articulation contacts the otic capsule, whereas a reduced mandibular condyle articulates with the lower jaw (Figs. 2A, B and 3AA, AB). The presence or absence of the squamosal is difficult to ascertain, but this is not unexpected, as this bone is barely identifiable even in extant specimens, ant the same applies to the epipterygoid.

#### Lower jaw

The right lower jaw (Figs. 2B and 4A–D) is complete and in articulation with the quadrate. The dentition is pleurodont and closely packed. The dentary (Fig. 4A–E), short and robust, bears eight teeth: the third tooth is the largest, whereas the fourth and the last ones are the smallest. The first tooth is not particularly enlarged, especially when compared to the third, which is clearly the largest. The symphysis shapes a marked angle with the ventral border of the dentary, which is roughly straight, only with a slightly convex central region. The subdental shelf has a high and rather rounded lingual surface (Fig. 4E). The Meckelian canal is open throughout all of its length (Fig. 4E), although it is posteriorly covered by a rather large splenial preserved in anatomical connection (Fig. 4A). A fused intramandibular septum (note that the homology of this element with those of anguids has been called into question, and it has been regarded as absent in other amphisbaenians [45])(Fig. 4E), covered by the anterior process of the coronoid and the anterior portion of the surangular/articular, closes the region between the posteroventral margin of the subdental shelf and the dorsal margin of the Meckelian canal. There are three large labial foramina situated at the levels between the first and second tooth, between the third and fourth, and under the sixth (Fig. 4B) Posteriorly, the dentary bears three different structures (Fig. 4B, E): a dorsally-positioned coronoid process, which is higher than wide and rather long; a surangular process that reaches a slightly more posterior position; and an angular process that marks the posterior-most point of the dentary. The postdentary region (Fig. 4A–D) is shorter than the dentary, but not as reduced as in other amphisbaenians, such as for example *Diplometopon* [44]. In contrast to most amphisbaenians [53], the postdentary bones do not constitute a compound bone (Fig. 4A–D). The splenial and the angular can be distinguished, but the articular and surangular are more difficult to separate in the CT scan, suggesting they probably represent a compound bone. The retroarticular process (Fig. 4A–D) is present, posteriorly directed, and not enlarged. The lower jaw has a dorsally-arched postdentary ventral region (Fig. 4A, B).

#### Vertebrae

Both cervical and trunk vertebrae are preserved (Fig. S1). They are all procoelous. The cervical segment is represented by four fragmentary vertebrae encrusted by a concretion that keeps them together (Fig. S1A–D). Their morphology is barely visible, but the referral to an amphisbaenian is supported by the following features: neural arch without neural spine; presence of a hint of prezygapophyseal processes; large and protruding synapophyses; and centra proportionally very short and narrow, slightly convex ventrally, and provided of a small hypapophysis. Moreover, the cervical vertebrae have neural arches with a truncated posterior tip and small paracotylar tubercles well separated from the large synapophyses. The remaining 45 isolated trunk vertebrae represent all trunk sectors and display a variety of morphologies and length (Fig. S1E–X). These vertebrae are rather large, with a centrum length (from the ventral edge of the cotyle to the posterior tip of the condyle) varying from 2.0 to 3.1 mm (Fig. S1E–X). Anterior trunk vertebrae are characterized by being wider and shorter than the posterior ones, with a taller neural arch and at least a hint of paracotylar foramina. Trunk vertebrae are otherwise characterized by the following morphology. In dorsal view, the prezygapophyses are prominent and developed in anterolateral direction; the prezygapophyseal facets are roundish or vaguely drop-shaped; the prezygapophyseal processes are small and stout (preserved only in few cases); the interzygapophyseal constriction is distinctly developed; the anterior edge of the neural arch is convex, whereas the posterior edge is notched (the median notch is delimited on both sides by a small convexity); the dorsal surface of the arch is thickened in the area surrounding such median notch, forming in some cases a ridge with the shape of an inverse V; the neural spine is absent, but a sort of sagittal ridge is developed in all the cases. In ventral view, the lower rim of the cotyle is regularly concave and posteriorly placed as compared to the dorsal rim; the most anterior trunk vertebrae show small paracotylar tubercles, which are regularly absent in the other vertebrae; the prezygapophyses are anterolaterally directed and show at least a hint of their process also in the cases in which they are not visible in dorsal view; the synapophyses are roundish and laterally protruding; the centrum is variably eleongated (especially in the most posterior vertebrae); the ventral surface of the centrum is rather flat and well delimited by straight or slightly concave lateral edges; two foramina pierce the ventral surface of the centrum in its anterior quarter; the cotyle surface is only minimally visible; the postzygapophyseal facets are elongated and drop-shaped. In lateral view, the neural spine is regularly absent; the dorsal edge of the neural arch can be variably concave – more concave in the anterior vertebrae, nearly straight in the most posterior ones – but is often flat close to the posterior edge (where the above-described V-shaped ridge is developed); the synapophyses are massive and globular; there are no lateral foramina; the boundary between the lateral and ventral surface is neat and corresponds to the ventral edge (there is no gradually sloping lateral surface); the dorsal edge of the dorsoventrally depressed condyle is placed much more anteriorly than the ventral edge. In anterior view, the cotyle is distinctly dorsoventrally depressed, oval with a nearly straight ventral rim; the neural canal is generally small and triangular; the dorsal edge of the neural arch is distinctly convex and in some cases tectiform and apically pointed; the zygosphene is regularly absent; the prezygapophyseal facets are distinctly tilted in dorsolateral direction; the synapophyses are massive and laterally protruding. In posterior view, the shape of the condyle matches that of the cotyle; the neural canal is wider than in anterior view; the posterior edge of the neural arch is markedly depressed and medially flat or nearly so; there is no evidence of zyganthra, but in some cases the dorsal surface of the medial edge of the postzygapophyseal facet delimits a small concavity along with the ventral surface of the neural arch; the ventral edge of the postzygapophyseal facets is tilted in dorsolateral direction.

## Results and Discussion

### The fossil record of Mediterranean worm lizards

Although amphisbaenians are abundant in Paleogene and Neogene localities from Europe, the usually fragmentary nature of the material hinders their identification. The basal phylogenetic position of the Blanidae among the Amphisbaenia points to a long fossil history for the former [3]–[8]. It is therefore possible that blanids were already present in Europe at least by the late Eocene, as suggested by some fossils attributed to *Blanosaurus* and less certainly to *Blanus* [55], [56]. Part of the Paleogene material previously referred to indeterminate amphisbaenids [55], [57], [58] is better attributed to indeterminate blanids [10], because the genus *Blanus* (to which similarities have been pointed) is no longer included in the former family [2]. The only exception regarding the incompleteness of the material is the articulated skeleton of *Cryptolacerta* from Messel (Germany), interpreted as a stem worm lizard [9].

Also on the basis of fragmentary remains, amphisbaenians other than blanids are present in Paleocene localities from Belgium and France in the form of *Polyodontobaena* and *Camptognathosaurus*, both included in the recently described family Polyodontobaenidae [56]. Moreover, uncertainties remain with regard to the attribution of several taxa. Thus, *Campinosaurus woutersi* – initially described as an anguimorph [59] and later argued to be an amphisbaenian [55], [10] – may not belong to this group, because the tooth count and morphology of the dentary both indicate scincoid affinities [60].

Even more problematic is the purported record in the early Eocene of France [10] of the North American genus *Anniealexandria*, with important paleobiogeographic implications. Such a referral is doubtful [60], because it is based on the presence of nine dentary teeth – a diagnostic character of this genus [10], which is seldom present in other genera. There is however some variability in the number of tooth positions among extant amphisbaenians. Thus, a count of nine dentary teeth has been also reported for several species of *Amphisbaena*, such as *Amphisbaena fuliginosa* [61], and it is also observable in the *Amphisbaena alba* figured in the literature [62], [63]. We further report a posteriorly located ninth small tooth in an extant *Blanus strauchi* dentary from Vic Siirt (Turkey) in the S. Bailon personal collection. In *Amphisbaena alba*, the replacement and the replaced tooth sometimes coexist [64], so that apparently increased counts (from typically eight to nine dentary teeth) might be related to the temporary retention of an old replaced tooth with the new, replacement one. It is also possible that ontogenetically older specimens, possessing longer dentaries, might accommodate a larger number of teeth. Although this should be confirmed through the study of ontogenetic series, dental counts are likely to be related to ontogenetic stage, so that adult, large squamate individuals would give more reliable tooth counts [65]. Unfortunately, the ontogenetic stage is usually difficult to ascertain from fossil specimens. Given these considerations, the referral of European material to the North-American genus *Annialexandria* might be incorrect [60], being alternatively attributable to an indeterminate amphisbaenian (?Blanidae) with nine dentary teeth.

The taxonomic status of other amphisbaenian genera from the Paleogene of Europe is also unclear. *Omoiotyphlops priscus*, from the Phosphorites du Quercy (Eocene or Oligocene from France) [66], is currently considered a nomen dubium, because it is based on few, undiagnostic vertebrae [55], [67]. *Louisamphisbaena ferox* from Grisolles (latest middle Eocene, France), in turn, is arguably a blanid [10], but the taxonomic validity of this genus is unclear, since the reported presence of a second curved tooth in the maxilla and the widely spaced teeth in the dentary do not enable a clear-cut distinction from *Blanus*. Moreover, no comparison to *Palaeoblanus tobieni* was made in the original description, despite sharing with the latter an enlarged first tooth – although *Louisamphisbaena* certainly lacks other characters of *Palaeoblanus*. Among the late Paleogene amphisbaenians, the monotypic blanid genus *Palaeoblanus* [50] is more clearly diagnosable than the other above-mentioned genera. This genus, originally described from the Miocene of Germany [50], has been also identified from the late Oligocene and Miocene of France, Germany, Italy and Spain [26]–[28], [68].

*Palaeoblanus* was not included in the Blanidae when the family was erected [2]. This is probably due to the poorly informative material referred to *Palaeoblanus* and the uncertainty of this distinctiveness of this genus from *Blanus*, rather than to any evidence against *Palaeoblanus* belonging to this family. Dentaries of *Palaeoblanus* possess a distinctly larger first tooth [26], [50], a more homogeneous and blunt dentition, and a more rounded symphysis than species of *Blanus*. On the basis of these features, we therefore support the distinct generic status of *Palaeoblanus*. At the same time, we support the ascription of *Palaeoblanus* to the Blanidae, thus representing the only extinct blanid genus recorded from the Neogene. A potential, currently unnamed, second species of *Palaeoblanus* has been reported from the Middle Miocene of Sandelzhausen (Germany) [68], based on the divergence of the lateral teeth. Such feature is however doubtful, because we found several specimens of *B*. *cinereus* (e.g., MDHC 156) with the same morphology – which is variable intraspecifically, and hence of no taxonomic value for diagnosing species. Moreover, the features purportedly justifying the referral of this material to *Palaeoblanus* – the proportion of the lateral teeth and the relatively larger size of the premaxillary foramina [68] – are insufficient to discount an alternative attribution to *Blanus* of the Saldenzhausen blanid material, which is best referred to as Blanidae indet. The French records at Mas de Got and Pech Desse [30] correspond to a large form with homodont, blunt teeth, most probably representing MP22 and MP28 records of *Palaeoblanus*.

Besides *Palaeoblanus tobieni*, only two extinct species of *Blanus* – *B*. *antiquus* and *B*. *gracilis*, from several German and Czech localities [29], [30] – are recognized in the Miocene. Even though similarities with the extant genus *Blanus* were noted, *Blanus gracilis* was originally attributed to a different genus, *Omoiotyphlops* [30], which is currently well established as a junior synonym of *Blanus* [69]. In fact, *B. gracilis* and *B*. *antiquus* have been considered synonymous by some authors [26], [69], in which case the nomen *B. gracilis* would have priority [31]. However, the smaller size, slenderer dentary and teeth, greater interdental space, and more heterodont dentition of *B*. *gracilis* compared to *B. antiquus* support their different species status. As it is evident in the corresponding drawings of Figure 6, *B*. *strauchi* and *B*. *gracilis* are much more similar to each other than to either *B*. *cinereus* or *B*. *antiquus*. Material from Sansan [69] clearly shows that two different forms are present in the same locality. Although similarities to *B*. *gracilis* and *B*. *antiquus* were noted for the smaller form, referred to *Blanus* sp. [69], in fact it shows greater similarities (mainly regarding the robust, heterodont and closely-packed dentition as well as the robustness of the dentary) with *B*. *mendezi* sp. nov. The slightly larger form (dentary length of 7 mm), left unassigned at the genus level, resembles instead *Palaeoblanus* (blunt crowns, rather homodont dentition, and rather rounded symphysis) [69].

There is no morphologic evidence that the above-mentioned extinct species of *Blanus* already belong to any of the several clades identified by molecular studies among the extant taxa [12], [49]. In contrast, fossil remains from Pliocene, Pleistocene and Holocene deposits of Western Europe (mainly Iberian Peninsula and Southern France) have been attributed to the extant *B*. *cinereus* [51], [70], [71]. Material from the latest Pliocene of Casablanca (Morocco), in turn, was referred to *Blanus* sp. [72]. Given that this locality is comprised within the present distribution range of *B*. *mettetali*, and very close to that of *B*. *tingitanus*, it is likely that these remains belong to one of the two extant species of the North-Western African clade. The same situation applies to the Pliocene record of an indeterminate amphisbaenian from Turkey [73], which might potentially belong to *B*. *strauchi* – or to an extinct species closely related to the latter from the Eastern clade.

### The amphisbaenian fossil record in the Iberian Peninsula

According to the available literature, amphisbaenian fossil remains from the Iberian Peninsula are not particularly abundant. However, if it is taken into account that Paleogene and Neogene herpetofaunas from this area remain understudied, this fact seems to be largely a sampling artifact that does not reflect a real absence.

With regard to the Paleogene, amphisbaenians have been described from the Early Eocene of Silveirinha [58], the late Eocene of Sossís [60] and the Oligocene of Montalbán [74]. Despite the rather fragmentary nature of the described remains, there is no clear evidence that these Paleogene specimens belong to an amphisbaenian group other than the Blanidae [60]. The Iberian Neogene record is substantially better than that from the Paleogene, although the material described so far is quite scarce. Miocene amphisbaenian remains have been reported from the Early Miocene of Córcoles, the Middle Miocene of Tarazona de Aragón, and the late Miocene of Can Missert, Los Valles de Fuentidueña, Viladecavalls, Can Llobateres and Bacochas, among other localities [27], [75], [76]. The possible presence of an amphisbaenian skull was reported decades ago from Viladecavalls [75], but the specimen was never described and it is currently lost (we were unable to locate it among the collections of the ICP). Amphisbaenian records from Iberian Plio-Pleistocene localities are more numerous [51], [70], [71], [77], including currently undescribed material [27].

In Iberia, *Palaeoblanus* has been reported from several Early to Middle Miocene localities (MN3–MN6) [27], but material has never been described or figured. The numerous blanid records from Spain (and France) reported in ref. [27], mainly based on undescribed material, show that Miocene remains are generally attributed to either *Blanus* sp. or *Palaeoblanus* sp., whereas the Plio-Pleistocene material is customarily attributed to *B*. *cinereus*. This higher taxonomic resolution for the more recent material is not attributable to a better knowledge (more complete preservation and/or higher number of recovered specimens), but related to the fact that researchers are more cautious when referring Miocene material to an extant species. The referral of Plio-Pleistocene Iberian remains to *B. cinereus* is further complicated by the recent description, mostly on molecular grounds, of the cryptic sibling species *B*. *mariae*, which would be morphologically very similar to *B. cinereus* [49]. Besides molecular differences, *B. mariae* has been reported to display a slightly larger size and some external morphologic differences compared to *B. cinereus*, but further research is required to confirm the distinct taxonomic status of the former as a distinct species instead of a subspecies of the latter – especially because it is unknown whether such differences are maintained or not in their contact zone [17]. The currently lack of osteological data for *B. mariae* seriously hinders the identification of Plio-Pleistocene Iberian blanids at the species level.

### General discussion

#### *Blanus mendezi* sp. Nov

The described skull, IPS60464, represents the most informative blanid fossil material ever described. Both the general configuration of the skull and the dental morphology of IPS60464 are in accordance with those of extant blanids, represented by the single extant genus *Blanus*. Similarities include: the tooth count (premaxilla: seven; maxilla: five; dentary: eight); the morphology, proportions and arrangement of skull bones (see above); and the shape and arrangement of the sutures – for a description of the cranial osteology of *B*. *cinereus* and *B*. *strauchi*, see ref. [47] and also below. Truncated nasals such as those displayed by IPS60464 (Fig. 3H, I) are the only diagnostic cranial features of blanids reported in the literature [2]. This character is unknown for fossil purported blanids, so that the ascription of isolated fossil material to this family has been mostly based on its overall similarity with the extant species of *Blanus*. Despite a recognized similarity to the genus *Blanus*, *Palaeoblanus* has not been formally referred to Blanidae – it was not mentioned in the erection of the family [2], and it was referred to the Amphisbaenidae by other authors [26], [27]. In contrast, we refer *Palaeoblanus* to the Blanidae on the basis of dentary morphologic similarities. IPS60464 differs from the extinct *Palaeoblanus* in lacking an enlarged first dentary tooth, and in displaying a heterodont and pointed dentition as well as a marked angle at the symphyseal level. These features allow an unambiguous attribution to the extant genus *Blanus*. IPS60464 therefore unambiguously confirms the presence of this genus in the European Miocene.

Moreover, as stated in the differential diagnosis above, the described cranial material differs from the two previously-described extinct species of this genus (*B. antiquus* and *B. gracilis*) – known from somewhat older localities [29], [30] – and also from extant *Blanus* spp. Besides the larger size of the former, differences include several dentognathic and/or cranial features (skull proportions, the shape of some sutures, and various morphologic details of the premaxilla, maxilla, frontals, nasals and dentary), thereby requiring the erection of the new species, *Blanus mendezi* sp. nov. (see diagnosis above). A more detailed evaluation of the taxonomic status of previously known fossil blanid species is precluded by their incomplete preservation. Thus, whereas tooth-bearing bones easily allow the discrimination between the monotypic genus *Paleoblanus* and *Blanus* spp., differences in this regard among *Blanus* species are subtler. Accordingly, the taxonomic status of both *B. antiquus* and *B. gracilis* should be subject to further scrutiny when more complete (cranial) remains become available, although they can be distinguished from *B. mendezi* on the basis of available evidence. With regard to extant species of this genus, a more detailed diagnosis of *B. mendezi* is also precluded – not by the morphology preserved in the holotype of the new species, but rather by the partial current knowledge on the osteology of living taxa. Thus, although the cranial morphology of extant amphisbaenians has been reported in several studies [47], [78], only that of *B. cinereus* and *B. strauchi* among extant blanids have been described in some detail [47]. In spite of this fact, the material described here sheds new light in the evolution of Mediterranean worm lizards from both phylogenetic and paleobiogeographic viewpoints.

### Comparisons with extant and extinct blanids

Many of the characters described in this paper for *Blanus mendezi* sp. nov. are unknown for other extinct blanids, thus being only directly comparable to extant members of the genus *Blanus* (Fig. 5). Among the four (or five) extant species of *Blanus*, only the osteology of *B*. *cinereus* and *B*. *strauchi* is partly known [47], and only the former has been previously reported from the fossil record. As a result, neither the inter- nor the intraspecific variability of osteological features within the genus *Blanus* is well known. Our survey of extant specimens suggests indeed that many cranial features display a considerable degree of intraspecific variability, supporting previous observations in this regard based on tooth-bearing bones [51]. For example, the maxillary tooth count in both *B. cinereus* and *B. strauchi* displays a range of variation between 3 and 5, so that the differences in this regard between the figured specimen of *B*. *cinereus* and *B*. *strauchi* (Fig. 5K, N) are not diagnostic at the species level. We also observed bilateral variability regarding the maxillary tooth count in one specimen of *B. strauchi* (MDHC 286), with the right maxilla having four teeth, and the left one just three. The specimens of *B. strauchi* MDHC 288, in turn, displays three teeth on the right maxilla and a protuberance on the posterior portion of the left maxilla, which indicates that a fourth tooth was about to erupt. Similarly, the number of premaxillary foramina seems to be variable, at least in *B. cinereus*, since we observed the presence of two foramina only in the left side of *B. strauchi* MDHC 287 (Fig. 5T); this fact contrasts with the usual condition of having a single foramen on each side (Figs. 3A, B and 5U). A similar bilateral variation has been noted for *B*. cf. *gracilis* [32], although in this case the double foramen was on the internal side. Intraspecific variability notwithstanding, cranial features are in general more taxonomically informative than postcranial ones for amphisbaenians; and, from a taxonomic viewpoint, the most informative cranial bones are the premaxilla and the frontals. We provide below detailed comparisons of *B. mendezi* with both extant and extinct blanids, by focusing on the most informative features.

#### Skull size

There are no published measurements of skull length for all extant blanid species, although the reported maximum head lengths can be used as a good proxy: 8.5 mm in *B*. *cinereus*, 7.9 mm in *B*. *mettetali*, and 7.3 mm in *B*. *tingitanus*, according to ref. [49]; a maximum of 8.5 mm in *B*. *cinereus*, a maximum of 9.6 mm in *B*. *mariae*, an average of 6 mm in *B*. *tingitanus*, and an average of ca. 5.6 mm in *B*. *mettetali*, according to ref. [49]. Direct measurements of maximum skull length of 8.0–8.1 mm for *B. cinereus* [47], [79] are only slightly smaller than external measurements, thus indicating only a slightly larger size for *B. strauchi*, on the basis of a maximum skull length of 8.5 mm for the former [47]. The skull length of 11.3 mm in *B. mendezi* therefore clearly shows that the new taxon was larger than all extant species of Mediterranean worm lizards – at least 15% longer than the largest skull reported [49]. Given the lack of complete skulls of extinct blanids other than *B. mendezi*, size comparisons between them must necessarily rely on the size of the dentaries. On this basis, the extinct *B. gracilis* would be roughly comparable in size to (only slightly smaller than) extant *B. cinereus* and *B. strauchi* (dentary length around 3.5 mm, maximum 4 mm in MDH 288 and 286), whereas the North-African *B*. *mettetali* and *B*. *tingitanus* would be even slightly smaller, based on reported skull size (see above). *B*. *antiquus* (dentary length up to 5 mm) and *B. mendezi* (6 mm) would be therefore larger than both *B. gracilis* and extant blanids. Some Plio-Pleistocene remains from the Iberian Peninsula have been sometimes reported to be larger than those of *B*. *cinereus*, the dentaries measuring 5–6.25 mm in the Early Pleistocene material from Illes Medes referred to *B. cinereus* [51]. Interestingly, some of the dentaries from Illes Medes display a small separation between the fifth and sixth teeth and, more rarely, between the seventh and the eighth ones [51]. These small gaps are present in *B*. *mendezi*, although it is unknown whether this character is shared with other members of the Western Mediterranean clade (it seems to be lacking in members of the Eastern Mediterranean clade). This might indicate that the material from Illes Medes does not belong to *B. cinereus*, and a possible referral to *B*. *mendezi* should be taken into account. Alternatively, if the material from Illes Medes belongs to *B*. *cinereus*, the species might have attained larger sizes in the past. In relation to this observation, a form similar in morphology to *B. cinereus*, but much larger, has been reported from the Late Miocene locality of Bacochas-1 (MN13) [80]. This large form might correspond to an additional record of *B*. *mendezi*, although further research would be required to confirm this possibility.

#### Nasal process of the premaxilla

An attribution of Miocene blanid remains to any of the extant clades is usually precluded by the incomplete preservation of the former. Among other features, the length of the nasal process of the premaxilla cannot be evaluated in most instances, because this bone is usually broken. Nonetheless, a complete premaxilla (DP FNSP 317) from the MN4 of Dolnice (Czech Republic) (figure 5 in ref. [30]) deserves in this regard a detailed comparison with the holotype of *Blanus mendezi*. The former specimen was referred to the Squamata indet., but on the basis of its morphology and size, it most likely belongs to *B. gracilis*, which has been reported on the basis of other remains from this very same locality [32]. Like *B. mendezi*, the premaxilla from Dolnice displays a long nasal process. In *B*. *mendezi*, such morphology is related to the fact that this process precludes the dorsal contact between the frontals for almost one-third of their length. The nasal process is generally shorter in *B*. *cinereus* than in *B*. *strauchi*, in spite of some variability among the specimens of the former examined while preparing this work (Fig. 5) and figured in the literature [47], [81]; see also Fig. S2. In some specimens, the nasal process of the premaxilla barely prevents the dorsal contact of the frontals, whereas in others their contact is precluded for a somewhat greater length. However, in none of the examined specimens the dorsal contact of the frontals is precluded to the same extent as in *B. mendezi*, thereby supporting the diagnostic validity of this feature.

#### Snout shape

Besides the length of the nasal process of the premaxilla per se, one of the few osteological features further enabling the distinction between extant species of *Blanus* is the projection of the snout relative to the lower jaws. In *B*. *strauchi*, the muzzle protrudes beyond the anterior-most level of the lower jaws and slightly curves downwards, whereas in *B*. *cinereus* the snout is not projecting [47], [16]. Such a projection of the snout in *B*. *strauchi* is also reflected in the shape of the premaxilla (Fig. 5U) – with an expansion at the anteroventral tip of the bone and an inward position of the teeth, which are situated relatively far from the anterior tip of the bone – thereby enabling the identification of *B*. *strauchi* from isolated premaxillae. In these regards, *B. mendezi* differs from *B*. *strauchi* and more closely resembles *B*. *cinereus* (and other previously-reported fossil blanid premaxillae), since in the former the anteroventral projection of the premaxilla is rather short, and the central tooth is almost aligned with the distalmost tip of this bone (Figs. 2A, 3A–C and 5T, U). The anteroventral projection of the premaxilla (reflecting the ventral projection of the snout over the retracted lower jaws) may the therefore interpreted as an autapomorphy of *B*. *strauchi*, given the fact that this feature is lacking in the rest of extant and fossil blanids.

#### Frontal and nasal length and shape

Some authors have noted the existence of two different extant morphs of *B*. *cinereus* in Spain [68]: one with strongly reduced lateral teeth and the typically robust nasal process of the premaxilla; and another one with lateral teeth not strongly-reduced and with a long and slender nasal process. The second morphotype is however based on a purported specimen of *B. cinereus* figured in ref. [29], which does not look amphisbaenian at all but resembles instead a lacertid premaxilla – i.e., with seven teeth equal in size and morphology, long and slender nasal process, and triangular posteroventral processes. An examination of previously figured blanids from the Iberian Peninsula [47], [78], however, shows that there are evident differences in frontal and nasal length and shape among several individuals (Fig. S2). In the specimen figured in ref. [47], the frontal is much longer and has a roughly rectangular shape, contrasting to the short and roughly square frontal in the specimen figured in ref. [78]; moreover, in the former the nasal is comparatively reduced, and the contribution of the dorsal margin of the maxilla to the dorsal region of the snout is restricted. It is uncertain whether these morphotypes might correspond, as speculated by some authors [68], to the two presumably cryptic species mainly distinguished on molecular grounds [49], [17] – i.e., *B. cinereus* (for the Central Iberian clade) and *B. mariae* (for the Southwestern Iberian clade). Their taxonomic status as a distinct (sub)species aside, the nominal taxa to be employed for each of these taxa is uncertain: first, because the lectotype designation for *B. cinereus* [49], based on a presumed syntype from the type series [82], is nomenclaturally incorrect [17]; second, because the exact localization of the type locality of this taxon (other than Portugal) is unknown [46], [11]; and third, because old names until recently considered junior synonyms of this species [11] should be examined, since depending on their type locality they might be senior synonyms of *B. mariae* [17].

A single frontal bone has been described from the European fossil record of amphisbaenians. This specimen, from the Early Pleistocene of Illes Medes (Spain) and attributed to *Blanus cinereus*, was incorrectly described as a nasal [51]; it actually corresponds to a frontal, which does not display significant differences compared to the extant *B*. *cinereus* (Fig. 5R, S) morphotype figured in ref. [47]. *Blanus mendezi* is more similar regarding frontal and nasal length and shape to these two specimens than to that figured in ref. [78]. The phylogenetic implications of this fact cannot be further evaluated at present, given the uncertain taxonomic status of *B. mariae* and the lack of osteological data for this taxon. In any case, the possibility cannot be discounted at present that the apparent variability in the length of the frontals in published specimens of *B. cinereus* is related to the presence of two different species. In this regard, the frontal of *B. mendezi* is clearly more elongated than that of both *B*. *strauchi* and *B*. *cinereus*, thus supporting the diagnostic value of this feature. This view is further strengthened by other distinctive features of the frontal of *B. mendezi*, namely: the marked and constantly-wide lateral facet for the articulation of the nasal, the maxilla and the prefrontal; the long anteromedial facet for the articulation of the premaxilla; the straight interfrontal suture; and the stronger interdigitation of the frontoparietal suture. Similarly, the nasal of the specimen figured in ref. [78] stands out by being much larger than those of *B*. *cinereus* and *B*. *mendezi*.

#### Maxilla

This element has not been described for either *P. tobieni* or *B*. *gracilis*. Compared to that of *B. antiquus* [29], the maxilla of *B. mendezi* displays a much longer and more pointed posteromedial process (orbital process). In addition, the medially directed rostral process of the maxilla (which makes this process to appear shorter) in *B. mendezi* clearly differs from that of *B*. *strauchi* (figure 5M, N), which is long and anteriorly directed. In this regard, *B*. *cinereus* (figure 5K, L) displays an intermediate morphology, which is however more similar to that of *B*. *mendezi* than to that of *B. strauchi*.

#### Sagittal crest

Although a sagittal crest on the parietal has been reported to be absent from *Blanus* [2], a faint crest can be observed on the anterior region of the parietal in both *B*. *cinereus* and *B*. *strauchi*, according to the specimens examined by us (Fig. 5U). A similar structure is apparently displayed by *B. mendezi* (Fig. 2A), which is most clearly seen in the CT sections than on the virtually reconstructed bone surface. Like in *B*. *strauchi* (Fig. 5Q), the faint crest of *B. mendezi* ends in a posteriorly situated protuberance (Fig. 2A). It therefore seems that, as in extant species of *Blanus*, in *B. mendezi* the sagittal crest is less developed than in other amphisbaenians, but not entirely lacking. The implications of this feature are not clear, because the presence of a sagittal crest is found in trogonophids, some amphisbaenids and some rhineurids [2]. Regarding amphisbaenids, the presence of a protuberance is variable among the different taxa [2]. We suggest that the sagittal crest and the presence of a protuberance on the parietal should be both regarded as present in *Blanus*.

#### Dentary and tooth counts

The extinct species of *Blanus* show some peculiarities in the dentary, although the taxonomic validity of such features (curvature and robustness of the teeth, as well as tooth count) is uncertain, due to the intraspecific variability shown in this regard by extant species. Compared to *B. mendezi*, the dentary of *B*. *gracilis* is smaller and slenderer, and displays straighter teeth and a greater interdental distance, thus more closely resembling *B*. *strauchi* (Figs. 5O, P and 6). In contrast, *B*. *antiquus* displays a more robust and homodont dentition, with the teeth almost contacting each other at their bases (Fig. 6). *Blanus cinereus* and *B*. *mendezi* would be intermediate regarding the robustness of their dentition (Fig. 6). The dentition of *B*. *strauchi* has been reported [29], [50] as being more slender and well spaced (approaching the condition of *B*. *gracilis*) than those of *B*. *cinereus* and *B*. *antiquus* (Fig. 6), which further holds when compared to *B*. *mendezi*.

A splenial in the dentary of *Blanus* has been variously reported as lacking [2] or present [48], [71]. We can confirm the presence of this bone in extant *Blanus strauchi*, *B*. *cinereus* and also the fossil *Blanus mendezi* (Fig. 4A).

Tooth counts are similarly of little help for distinguishing *B. mendezi*, not only regarding the premaxilla and maxilla, but also with regard to the dentary (Figs. 2D and 6). Thus, *B. mendezi* displays seven teeth in the premaxilla, as extant species of *Blanus* [32], and five teeth in the maxilla; extant *Blanus* display 3–5 maxillary teeth [29], the second one being the largest when five teeth are present, as in *B. mendezi*. With regard to the dentary, the 8 teeth present in *B. mendezi* fit well with the range of 7–8 teeth usually reported for extant *Blanus* [29]. This feature is in fact quite variable, since we have observed the presence of nine teeth in at least one specimen of *B*. *strauchi* in which the posterior-most tooth is extremely small, but present in both dentaries.

#### Vertebrae

The vertebrae of *B*. *cinereus* and *B*. *strauchi* are indistinguishable (Fig. 5A–J). In fact, amphisbaenian vertebrae are generally not diagnostic at the genus or even the family levels – blanid and amphisbaenid vertebrae being difficult to differentiate. The abundant amphisbaenian vertebrae recovered from the type locality of *B. mendezi* are attributed to this taxon, given the lack of non-blanid worm lizards in the European Neogene fossil record [27] and the fact that they are consistent in size with the holotype of the new species – being, like the skull, larger than those of other extinct and extant blanids. In *Blanus*, the length of the centrum usually ranges from 1.5 to 2.0 mm (MDHC 187 for *B*. *cinereus* and MDHC 286, 287, 288 for *B*. *strauchi*), whereas those of *B. mendezi* reach a maximum length of 3.1 mm. This length is exceeded by the vertebrae from the collection of Sansan [69], in which vertebrae reach a maximum length of 4 mm. However, as reported above, two forms are present at Sansan on the basis of size of the dentaries. A more abundant form, smaller in size, is referred to *Blanus* sp., whereas the other, much more scarce and much larger, probably belongs to *Palaeoblanus*.

With regard to morphology, the cervical vertebrae of *B. mendezi* are very similar to those of extant *B*. *cinereus* and *B*. *strauchi* (Fig. S1). The vertebrae of *B. mendezi*, however, can be distinguished from those of both *B. cinereus* and *B. strauchi* not only by the larger size of the former, but also by the presence of paracotylar tubercles in the cervical and anterior trunk vertebrae. This fact further supports the distinct species status of *B. mendezi*, although additional extant blanid specimens should be examined to completely discount that this feature is intraspecifically variable and/or size-related. The same structure might be present in *B. gracilis*, where a conspicuous protuberance has been reported close to the margin of the cotyle and ventrally to the synapophysis [30]. This structure has not been described in any other blanid, and is certainly absent from accessed extant material (*B*. *cinereus* and *B*. *strauchi*) and fossil *Palaeoblanus* from the MN13 of Gargano (Italy) [28].

### Paleobiogeography and phylogeny

The extensive similarities in cranial morphology between *B. mendezi* and extant Mediterranean worm lizards indicate that the genus *Blanus* is not only conservative regarding the tooth-bearing bones and vertebrae – as shown by the identification of *Blanus*-like forms already in the Eocene [10], [56] – but also regarding the rest of skull bones and lower jaw. Contrasting with its present disjunct and restricted, almost relictual distribution [16], [82], the genus *Blanus* was much more widely distributed across Europe in the past. The oldest report of this genus dates back to the Late Eocene of England [83], although it is based on very fragmentary remains that do not allow an unambiguous attribution to *Blanus* [10], [50]. The amphisbaenian Paleogene European record has recently improved [10], [56], so that when better known it might shed further light on the initial steps of blanid evolution. Meanwhile, IPS60464 provides key information for discussing the more recent evolution of this family.

*Blanus* was widely distributed in Europe during the Miocene, subsequently showing a progressive reduction of its range, until it became restricted to the Mediterranean shores in the Pliocene [18]. In the tree depicted in Fig. 6 we synthesize currently available evidence for blanid evolution based on molecular, paleontological and biogeographic data. Blanids would have diverged from other amphisbaenians before 25 Ma [12], [49], and possibly much earlier if at least some of the Eocene forms belong to the family [10], [56]. *Palaeoblanus* displays homodont dentaries, with blunt, large and robust cusps, and also bears a characteristic enlarged first tooth and a rounded symphysis. Among these features, the first enlarged tooth, the blunt cusps and the rounded symphysis characterize *Palaeoblanus* alone, whereas homodonty, large size and robustness are characters shared with *Blanus antiquus*. According to our phylogenetic hypothesis, the non-enlarged first tooth, the pointed crowns, and the well-marked symphyseal angle would be synapomorphies of the *Blanus* clade. All the species of this genus, to the exception of *B*. *antiquus*, further share a marked heterodonty in their dentaries, best expressed by the evident size reduction of the fourth tooth. Among these species, the Western clade is characterized by the robustness of its dentitions and dentaries, whereas the Eastern clade possesses much slenderer dentaries with well-spaced, gracile teeth.

The Eastern clade is formed by the extinct *B*. *gracilis* and the extant *B*. *strauchi*. Dentaries in both forms can be barely differentiated, but the premaxilla of *B*. *strauchi* is easily distinguished from that of *B*. *gracilis* (and from all other species for which this bone is known) because of its ventrally projected proximal tip. The Western clade, in turn, is constituted by the large and robust fossil form *B*. *mendezi*, together with four extant species that, according to molecular analyses, can be further divided into an Iberian clade (*B*. *cinereus* +*B*. *mariae*) and a North-Western African clade (*B*. *mettetali* +*B*. *tingitanus*). Many morphologic skull characters distinguish *B*. *mendezi* from *B*. *cinereus* (see Differential Diagnosis above). However, given that the morphology of the remaining members of the Western clade is barely known, it is difficult to discern whether the features of *B*. *mendezi* characterize it in front of the rest of the Western clade, or merely in front of part of its members (e.g., the Iberian clade). However, all of the Western members share a much smaller size, and reported external morphologic differences from *B*. *cinereus* are minimal (*B*. *tingitanus*, *B*. *mettetali* and *B*. *mariae* were formerly included in *B*. *cinereus*). Therefore, we consider it more likely that the species of this clade diverged late, most probably well after the split of *B*. *mendezi*. This interpretation also fits well with the scenario proposed in ref. [12], in which the divergence between the Eastern and Western Mediterranean clades would have occurred 8–9 Ma due to the opening of the Betic corridor. Alternatively, if a strict Iberian clade (with *B*. *cinereus* +*B*. *mariae* +*B*. *mendezi*) would be recognized in the future based on morphologic evidence, then the minimum divergence date between the Iberian and African clades should be moved backwards from 8–9 Ma [12] to at least 11.6 Ma.

The geographic and temporal distribution of fossil worm lizards is congruent with the phylogenetic relationships proposed in figure 6 for the Blanidae. *Palaeoblanus* and *B*. *antiquus* have the oldest records, and are widespread in Central and Western Europe. Given that the late Paleogene and early Neogene record in Eastern Europe is quite poor, it cannot be discounted that their distribution actually reached Eastern Europe (where the records would be lacking due to a sampling artifact). The record of *B*. *gracilis* at Dolnice (MN4b, 14–16 Ma) would mark the minimum divergence time between the Eastern and Western clades. The long ghost lineage of *B*. *strauchi* could be easily explained by the poor fossil record from the Neogene of Eastern-most Europe. The Middle Miocene age of *B. mendezi* and the slightly older age of *B*. *gracilis* are both congruent with molecular estimates of the divergence time between *B. strauchi* and Western *Blanus* to the Early Miocene (16.5 Ma). However, this is not a maximum divergence time – contra ref. [12] – but a minimum one. It has been proposed that the divergence between the Western and Eastern Mediterranean clades would have been caused by an extinction of the Central European populations [16], so that the split between these two clades could not be older than the youngest Central European record, i.e., Middle Miocene [12]. However, this is contradicted by the fact that *B*. *antiquus*, from Central Europe, is contemporaneous with *B*. *gracilis*, which is a member of the Eastern Mediterranean clade (figure 6). This fact indicates that the divergence between the Western and Eastern clades predates the youngest Central European record, so that their split would not be related to the extinction of Central European populations (which would have persisted afterwards).

After the divergence of the Iberian and North Western African subclades, the genus *Blanus* was much more widely distributed than nowadays throughout the circum-Mediterranean region [18] – being recorded from areas currently not inhabited by worm lizards. These areas include the Balkans, the Italian Peninsula and concomitant islands, where they survived until the Late Pleistocene [84], [85], as well as the Balearic Islands, which blanids probably reached during the Messinian Salinity Crisis by the latest Miocene [86]. More complete (cranial) fossil remains of Late Miocene and Pliocene *Blanus*, in particular from those areas where they are currently absent, would be required to further test the molecular divergence times between the various species of the Western clade. *Blanus mendezi*, in any case, represents the oldest record of the Western Mediterranean clade, slightly postdating the oldest record of the Eastern Mediterranean clade. Unlike previously calibration points employed by molecular studies [12], [49], the evidence provided by *B. mendezi* is not based on paleobiogeographic assumptions, but on fossil evidence, thus being of greatest significance for further refining molecular phylogenetic studies in the future. Furthermore, *B. mendezi* will serve as a solid comparative reference for deciphering the internal phylogeny of the genus *Blanus* as well as their position among amphisbaenians and its most likely sister taxon – especially once the cranial osteology of the extant species of this genus is known in greater detail.

## Supporting Information

Figure S1 **Selected vertebrae (paratypes) of** ***Blanus mendezi*** **sp. nov.** (A–D) Four cervical vertebrae in anatomical connection (IPS63989), in left lateral (A), right lateral (B), dorsal (C), and ventral (D) views. (E–X) Dorsal vertebrae (IPS63990–IPS63993), in dorsal (E, J, O, T), ventral (F, K, P, U), left lateral (L), right lateral (B, G, Q, V), cranial (H, M, R, W) and caudal (I, N, S, X) views. Arrows in D and U indicate paracotylar tubercles. Scale bar equals 2 mm.
(TIF)

Figure S2 **Schematic drawings of the cranium of** ***Blanus mendezi*** **sp. nov., in dorsal view, compared to those of** ***Blanus cinereus*** **.** (A) *B*. *mendezi*, based on the virtual model of the holotype (IPS60464). (B–C) *B*. *cinereus*, redrawn from ref. [44] (B) and ref. [78] (C).
(TIF)

Video S1 **IPS60464, video showing the digital removal of the covering crust and infilling matrix, and rotation of the resulting virtual model.**
(MP4)
