# Supplementary figures and images for: An Amphisbaenian Skull from the European Miocene and the Evolution of Mediterranean Worm Lizards
Source: PLoS One. 2014 Jun 4;9(6):e98082. doi: 10.1371/journal.pone.0098082 (PMC4045672; doi:10.1371/journal.pone.0098082)

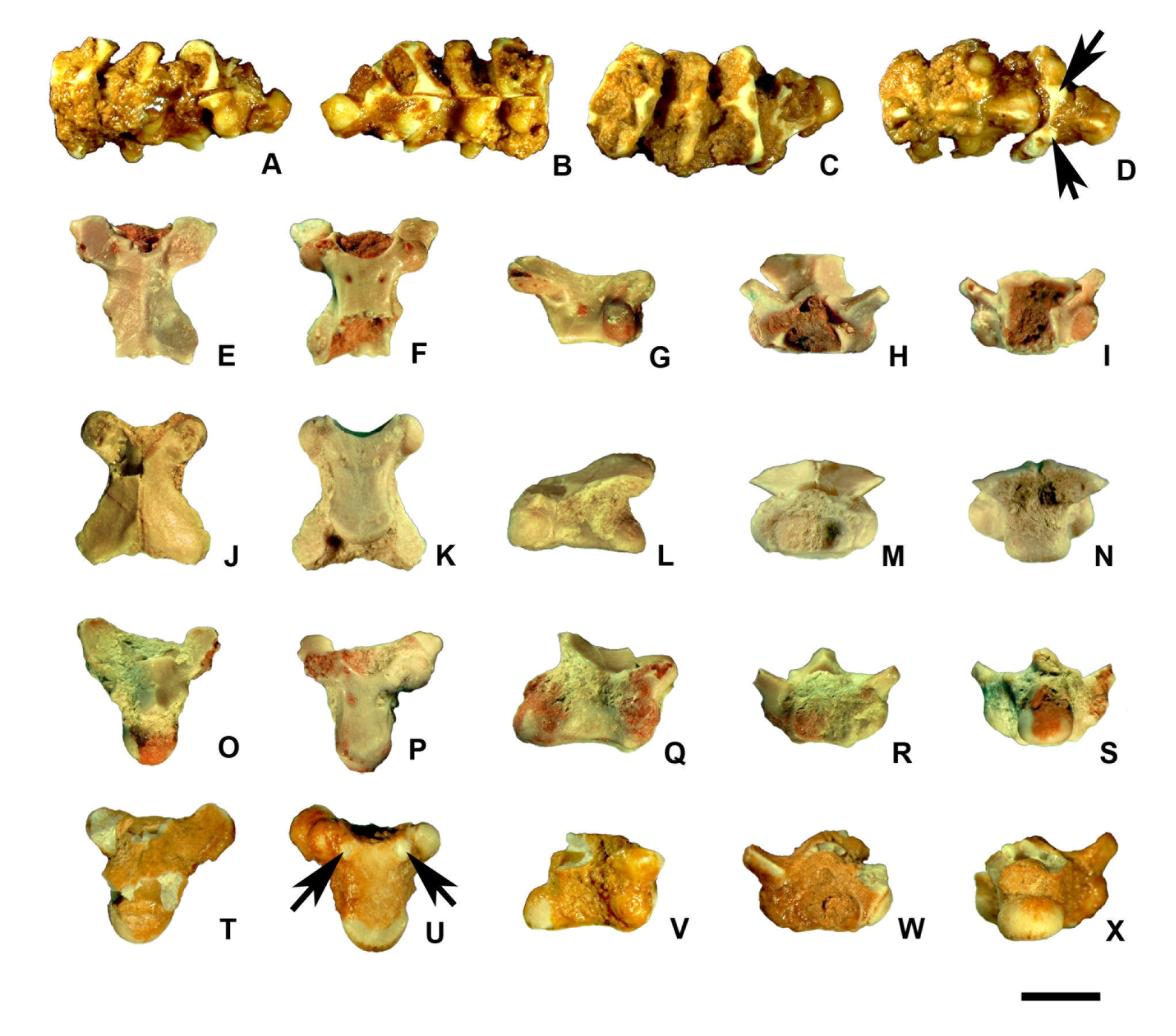

Supplement: Figure S1 — Selected vertebrae (paratypes) of Blanus mendezi sp. nov. (A–D) Four cervical vertebrae in anatomical connection (IPS63989), in left lateral (A), right lateral (B), dorsal (C), and ventral (D) views. (E–X) Dorsal vertebrae (IPS63990–IPS63993), in dorsal (E, J, O, T), ventral (F, K, P, U), left lateral (L), right lateral (B, G, Q, V), cranial (H, M, R, W) and caudal (I, N, S, X) views. Arrows in D and U indicate paracotylar tubercles. Scale bar equals 2 mm. (TIF) [file pone.0098082.s001.tif]

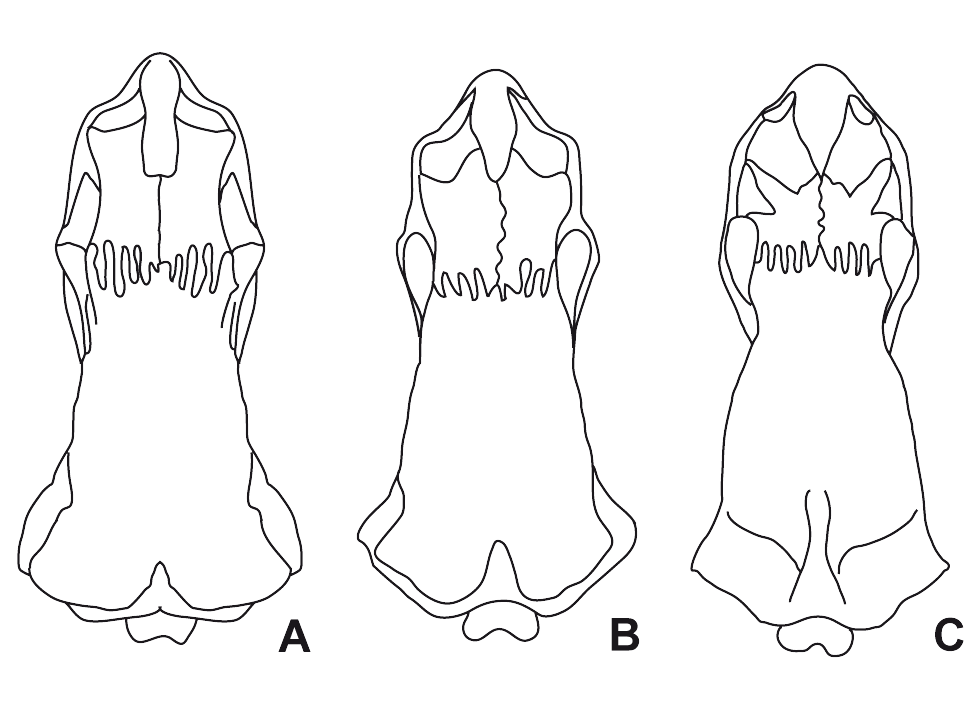

Supplement: Figure S2 — Schematic drawings of the cranium of Blanus mendezi sp. nov., in dorsal view, compared to those of Blanus cinereus . (A) B. mendezi, based on the virtual model of the holotype (IPS60464). (B–C) B. cinereus, redrawn from ref. [44] (B) and ref. [78] (C). (TIF) [file pone.0098082.s002.tif]
